# Relationship between preoperative plasma fibrinogen and prognosis in patients with non-metastatic gastric cancer: a systematic review and meta-analysis

**DOI:** 10.3389/fonc.2025.1624602

**Published:** 2025-12-04

**Authors:** Yan-Rong Lei, Lu Lv, Chi Lu

**Affiliations:** Department of Oncology, The Central Hospital of Wuhan, Tongji Medical College, Huazhong University of Science and Technology, Wuhan, Hubei, China

**Keywords:** gastric cancer, fibrinogen, prognosis, overall survival, recurrence-free survival, systematic review, meta-analysis

## Abstract

**Background:**

Preoperative plasma fibrinogen (Fib) is a potential prognostic marker for various cancers, including gastric cancer. This systematic review and meta-analysis aimed to investigate the relationship between preoperative fibrinogen levels and prognosis in patients with non-metastatic gastric cancer patients.

**Methods:**

This meta-analysis was conducted in accordance with the PRISMA guidelines. A comprehensive literature search of PubMed, Embase, Web of Science, and Cochrane Library was performed up to May 26, 2025, without language or date restrictions. Eligible studies reported multivariate-adjusted hazard ratios (HRs) and 95% confidence intervals (CIs) for overall survival (OS) and recurrence-free survival (RFS) in relation to preoperative fibrinogen levels. Subgroup and sensitivity analyses were performed, and study quality was assessed using the Newcastle-Ottawa Scale (NOS).

**Results:**

A total of 8 retrospective studies involving 4,281 patients were included. Pooled analysis revealed that elevated fibrinogen levels were significantly associated with poorer OS (HR = 1.56, 95% CI: 1.20–1.93, P < 0.001; I² = 57.0%) and RFS (HR = 2.08, 95% CI: 1.33–2.82, P < 0.001; I² = 0%). Subgroup analyses confirmed consistent associations across age, sex, tumor stage, geographic region, and fibrinogen cut-off values. No significant publication bias was detected.

**Conclusions:**

Elevated preoperative fibrinogen levels are significantly associated with worse overall and recurrence-free survival in patients with non-metastatic gastric cancer, indicating its potential utility as a prognostic biomarker. Given the limited data on RFS, further large-scale prospective studies are needed to validate these findings and support its integration into individualized risk stratification models.

## 1 Introduction

Gastric cancer (GC) remains a major global health challenge, ranking as the fifth most common malignancy and the fourth leading cause of cancer-related death worldwide (1). Despite advances in diagnosis and therapy, the overall prognosis of GC remains poor, particularly for patients diagnosed at advanced stages. Even among those with non-metastatic disease undergoing curative resection, recurrence is frequent and long-term survival remains unsatisfactory (2). These challenges highlight the urgent need for reliable prognostic indicators to improve early detection, risk stratification, and personalized treatment strategies (3). Preoperative identification of robust prognostic biomarkers is crucial for accurate risk assessment, individualized therapeutic planning, and effective postoperative surveillance (4). Conventional tumor markers such as carcinoembryonic antigen (CEA) and carbohydrate antigen 19-9 (CA19-9) are widely used but limited by low sensitivity and specificity, particularly in early-stage disease (5). This limitation has prompted increasing interest in alternative biomarkers that are accessible, cost-effective, and capable of reflecting tumor burden, biological behavior, and host systemic responses to malignancy (6).

Cancer-associated inflammation and coagulation abnormalities play pivotal roles in tumor initiation, invasion, and metastasis. Among coagulation-related proteins, plasma fibrinogen (Fib) has gained attention as a promising prognostic biomarker (7, 8). As a key glycoprotein in hemostasis and a major acute-phase reactant, fibrinogen is upregulated by pro-inflammatory cytokines released from tumor cells and the surrounding microenvironment (9). This cytokine-driven elevation contributes to a hypercoagulable state characterized by increased thrombin generation, fibrin deposition, and platelet aggregation, which collectively promote tumor progression and immune evasion (10). Beyond its role in coagulation, fibrinogen is increasingly recognized as a critical modulator of immune and inflammatory pathways that influence cancer prognosis. It interacts with immune cells, including macrophages and lymphocytes, and regulates cytokine-mediated signaling, particularly involving interleukin-6 (IL-6) and interleukin-8 (IL-8), which promote tumor-associated inflammation and immune suppression (11). Moreover, fibrinogen facilitates angiogenesis and mast cell activation within the tumor microenvironment, both of which contribute to neovascularization, extracellular matrix remodeling, and tumor invasion (12). Elevated levels of coagulation components such as platelets, fibrinogen, and D-dimer have been associated with increased metastatic potential and poor prognosis in various solid tumors. These findings provide a strong rationale for further investigation into the prognostic significance of preoperative fibrinogen levels in patients with non-metastatic gastric cancer (13).

In recent years, elevated preoperative fibrinogen levels have been associated with poor prognosis in several malignancies, including hepatocellular carcinoma, pancreatic cancer, colorectal cancer, renal cell carcinoma, and upper tract urothelial carcinoma (14, 15). These findings suggest that fibrinogen may serve as a clinically relevant prognostic biomarker. However, its prognostic value in gastric cancer remains inconclusive. For example, Yu et al. (16) reported that fibrinogen levels were significantly elevated in gastric cancer patients and positively correlated with tumor stage. In contrast, Wakatsuki et al. (17) found no significant association between preoperative fibrinogen levels and survival outcomes in patients with gastric cancer. This inconsistency in the literature underscores a critical gap in understanding the prognostic role of preoperative fibrinogen in non-metastatic gastric cancer. Such discrepancies may be attributed to variations in study design, patient selection, and fibrinogen measurement or threshold definitions, highlighting a lack of standardized evidence synthesis. This inconsistency underscores a critical gap in understanding the prognostic role of preoperative fibrinogen in non-metastatic gastric cancer. Therefore, we conducted a systematic review and meta-analysis focusing on patients with non-metastatic gastric cancer to integrate available evidence and quantitatively evaluate the association between preoperative fibrinogen levels and survival outcomes. By synthesizing current data, we aim to clarify the prognostic significance of fibrinogen, support its potential clinical utility, and inform future biomarker-driven strategies for individualized risk assessment in gastric cancer.

## 2 Materials and methods

### 2.1 Search strategy

During the systematic review process and subsequent reporting of our results, we adhered to the Preferred Reporting Items for Systematic Reviews and Meta-Analyses (PRISMA) guidelines (18). Four electronic databases, PubMed, Embase, Web of Science, and Cochrane Library, were searched on May 26, 2025, and no time limitation was applied. An updated manual search was conducted on November 5, 2025, and no additional eligible studies meeting the inclusion criteria were identified. The vocabulary and syntax were adapted according to the database. Search terms were related to “gastric cancer,” “fibrinogen,” and “prognosis,” and were adapted to the specific syntax and indexing of each database. Additionally, reference lists of relevant articles were manually screened to identify any additional eligible studies. The detailed search strategies for each database are provided in **Supplementary Table S1** to ensure transparency and reproducibility.

### 2.2 Inclusion criteria

Studies included in this systematic review were required to meet the following criteria:1) investigations evaluating the association between plasma fibrinogen (Fib) levels and the prognosis of non-metastatic gastric cancer; 2) studies reporting a clearly defined fibrinogen cut-off value; 3) outcome measures including overall survival (OS) and/or recurrence-free survival (RFS); 4) studies providing hazard ratios (HRs) with 95% confidence intervals (CIs) derived from multivariate survival analyses; and 5) for duplicate publications based on the same population, only the study with the largest sample size or the most recent data was included.

The exclusion criteria were as follows:1) studies involving pediatric patients or those with metastatic gastric cancer; 2) studies of low methodological quality or lacking original data; and 3) case reports, commentaries, expert opinions, and narrative reviews.

### 2.3 Data extraction

Data acquisition and selection were independently performed by two investigators. Titles and abstracts were screened to exclude clearly irrelevant studies, followed by full-text evaluations to determine eligibility based on the predefined inclusion and exclusion criteria. Any discrepancies in study selection or data extraction were resolved through discussion with a third reviewer. The following information was extracted from each eligible study: first author, country, year of publication, study design, sample size, patient age, follow-up duration, fibrinogen cut-off value, rationale for cut-off selection, tumor stage, and other relevant clinicopathological characteristics. Additionally, hazard ratios (HRs) and 95% confidence intervals (CIs) were extracted from both univariate and multivariate survival analyses, with preference given to multivariate results when available. Subgroup analyses were conducted according to key stratification variables. For studies that did not directly report HRs and 95% CIs, survival data were extracted from Kaplan–Meier curves using Engauge Digitizer (version 4.1). The extracted data were then used to estimate HRs and their corresponding 95% CIs following the methods proposed by Tierney et al. (19) and Parmar et al. (20). Two investigators independently performed the data extraction from survival curves, and any disagreements were resolved through consensus. Although this indirect method may introduce minor estimation bias, it is widely accepted in meta-analyses of time-to-event outcomes and provides a reasonable approximation of effect sizes when direct summary statistics are unavailable. To enhance reproducibility, data extraction consistency was verified by cross-checking a subset of studies, and discrepancies were below 5%, indicating high inter-reviewer reliability (21).

### 2.4 Quality assessment

The methodological quality of the studies included in this meta-analysis was rigorously evaluated by two independent reviewers using the Newcastle-Ottawa Scale (NOS) (22). The NOS is a widely recognized instrument for assessing the quality of non-randomized studies, particularly observational studies, included in systematic reviews and meta-analyses. It evaluates studies across three domains: selection, comparability, and outcome, thereby identifying potential sources of bias. Each study was assigned a score ranging from 0 to 9, with scores of 0–3 indicating low quality, 4–6 indicating moderate quality, and 7–9 indicating high quality. Any disagreements between reviewers were resolved through discussion or consultation with a third reviewer.

### 2.5 Statistical analyses

The degree of heterogeneity among the included studies was assessed using the chi-square test and quantified with the I² statistic. An I² value of 0% indicated no apparent heterogeneity, whereas values exceeding 50% were considered to represent substantial heterogeneity. In cases of significant heterogeneity (I² > 50%), a random-effects model was applied to estimate the pooled effect size, accounting for both within- and between-study variability. Conversely, when heterogeneity was not significant (I² ≤ 50%), a fixed-effects model was used, which considered only within-study variance. Sensitivity analyses were conducted to evaluate the robustness of the results and to identify the influence of individual studies on the pooled estimates by sequentially omitting each study and recalculating the combined effect size. The stability of the findings was confirmed if the recalculated estimates remained within the 95% confidence interval of the original pooled effect. Publication bias was assessed visually using funnel plot symmetry and statistically using Egger’s regression test. All statistical analyses were two-sided, and a P-value < 0.05 was considered statistically significant. Data analyses were performed using Stata version 17.0 (StataCorp, College Station, TX, USA).

## 3 Results

### 3.1 Search results and study selection

**Figure 1:**
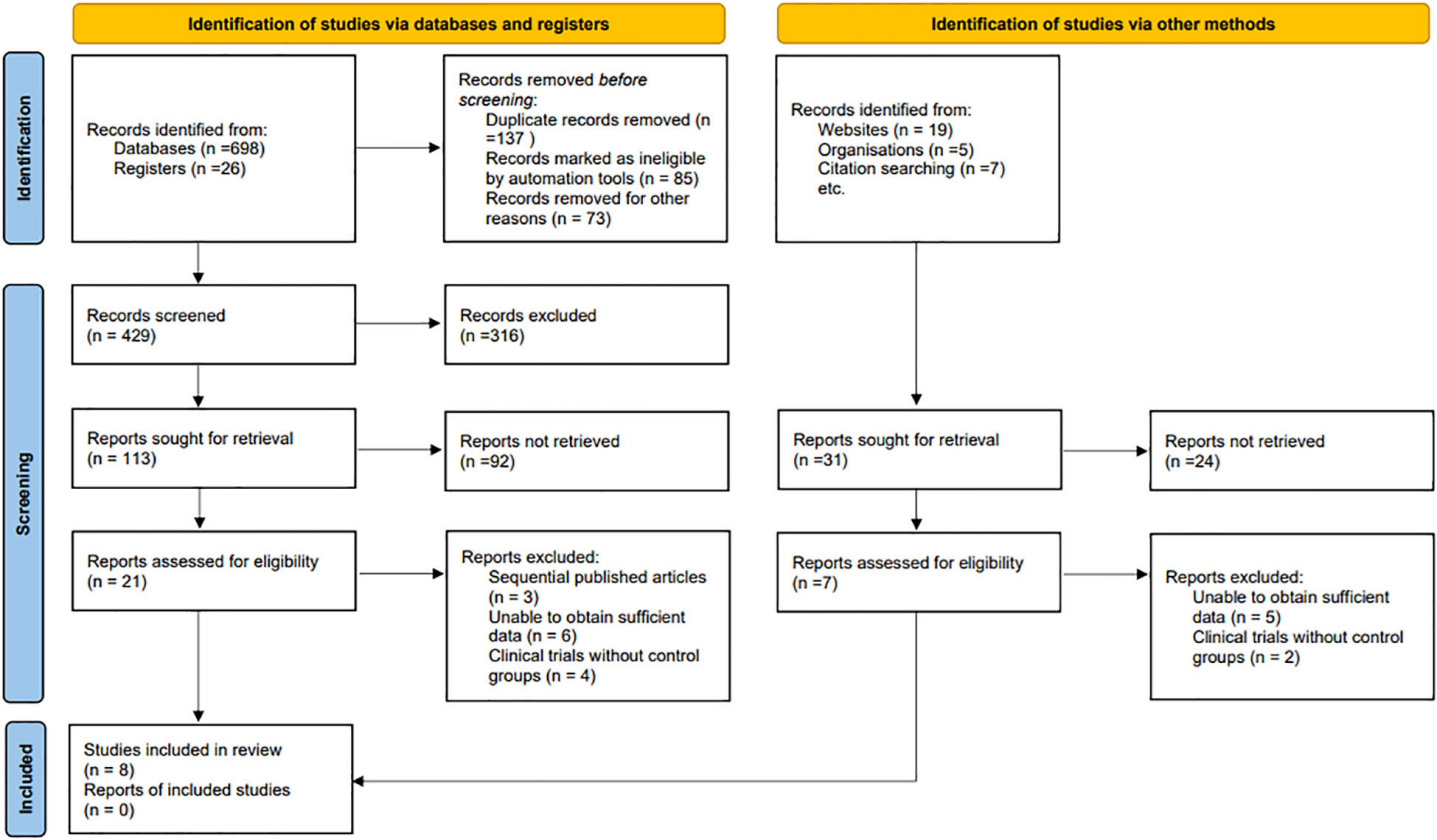
Selection process of included studies.

From the initial search of electronic databases, 724 related studies were found. After removing repetitive literature, reading titles and abstracts, and screening strictly according to the inclusion and exclusion criteria, 21 related studies were obtained and 13 were excluded from further reading. Finally, 8 articles were included (17, 23–29). The literature screening process and the results are shown in **Figure 1**.

### 3.2 Study characteristics

The characteristics of the included studies are summarized in **Table 1**. The studies included in our meta-analysis spanned 2000 to 2019 and originated from two countries, Japan and China. All studies were retrospective in nature. The total sample size across these studies was quite substantial, with the number of patients varying from as low as 123 (93 males and 30 females) to as high as 1,293 (882 males and 411 females). Most of the studies investigated patients across all stages of the disease (I-IV), although a few focused specifically on stages II-III. The key outcome measures were OS and RFS. These studies employed different cutoff values for fibrinogen, ranging from 3.30 to 4.00 g/L. The method for determining the cutoff value was mostly through ROC analysis, while some studies used the X-tile method or did not specify their method. The follow-up duration also varied, with some studies offering a short span of one month to others extending the follow-up to over 123 months.

**Table 1:** Characteristics of studies included in the meta-analysis.

Author	Year	Country	Study type	Period	No. of patients (M/F)	Outcome measures	Fibrinogen cut-off value (g/L)	Method for cut-off value	Follow-up duration (months)	Stages
Zhao et al.	2019	China	R	2002-2012	592/250	OS	3.20	X-tile	>12	I-IV
Wakatsuki et al.	2018	Japan	R	2001-2006	137/45	OS/RFS	2.60	ROC	>12	I-III
Liu et al.	2018	China	R	2000-2012	882/411	OS	4.00	–	35	I-III
Kanda et al.	2017	Japan	R	2003-2016	93/30	OS	4.00	–	1-60	II-III
Zhang et al.	2017	China	R	2011-2013	261/99	OS	3.30	–	1-36	II-III
Arigami et al.	2016	Japan	R	2000-2011	179/96	OS	3.05	ROC	1-123	I-IV
Suzuki et al.	2016	Japan	R	2008-2013	212/103	OS	3.50	ROC	28	I-IV
Yamamoto et al.	2016	Japan	R	2001-2011	436/173	OS/RFS	3.50	ROC	55	I-IV

“M/F” denotes the number of male and female patients, respectively. “R” refers to Retrospective, “OS” refers to overall survival, “RFS” to recurrence-free survival, and “ROC” to receiver operating characteristic. The “Period” column indicates the time frame of each study. In the “Stages” column, Roman numerals denote the stage of gastric cancer in the studied patients.

### 3.3 Results of quality assessment

We utilized NOS to evaluate the methodological rigor of each study involved in our meta-analysis. Across the board, two studies attained a score of 7, while three studies each received scores of 8 and 9. It should be noted that no studies have incorporated blinding methods or made provisions for allocation concealment. Moreover, there were no indications of funding bias in any of the included studies. There were no instances of incomplete outcome data, premature termination biases, or imbalances in baseline data. A comprehensive summary of the risk of bias and the corresponding ratios is presented in **Table 2**.

**Table 2:** he quality assessment according to Newcastle-Ottawa scale.

Study	Selection				Comparability	Outcome			Total score
	Representativeness of the exposed cohort	Selection of the non -exposed cohort	Ascertainment of exposure	Demonstration that outcome	Comparability of cohorts	Assessment of outcome	Was follow-up long enough	Adequacy of follow up of cohorts	
Zhao et al.	★	★	★	★	★★	★	★	★	7
Wakatsuki et al.		★	★	★	★★	★	★	★	8
Liu et al.	★	★	★	★	★★	★	★	★	9
Kanda et al.	★	★	★	★	★★	★		★	8
Zhang et al.	★		★	★	★★		★	★	9
Arigami et al.	★	★	★	★	★	★	★	★	8
Suzuki et al.	★	★	★	★	★★	★	★	★	7
Yamamoto et al.	★		★	★	★	★	★	★	9

A star (★) was assigned according to the Newcastle-Ottawa Scale (NOS) criteria.

### 3.4 Preoperative fibrinogen levels and OS in gastric cancer

**Figure 2:**
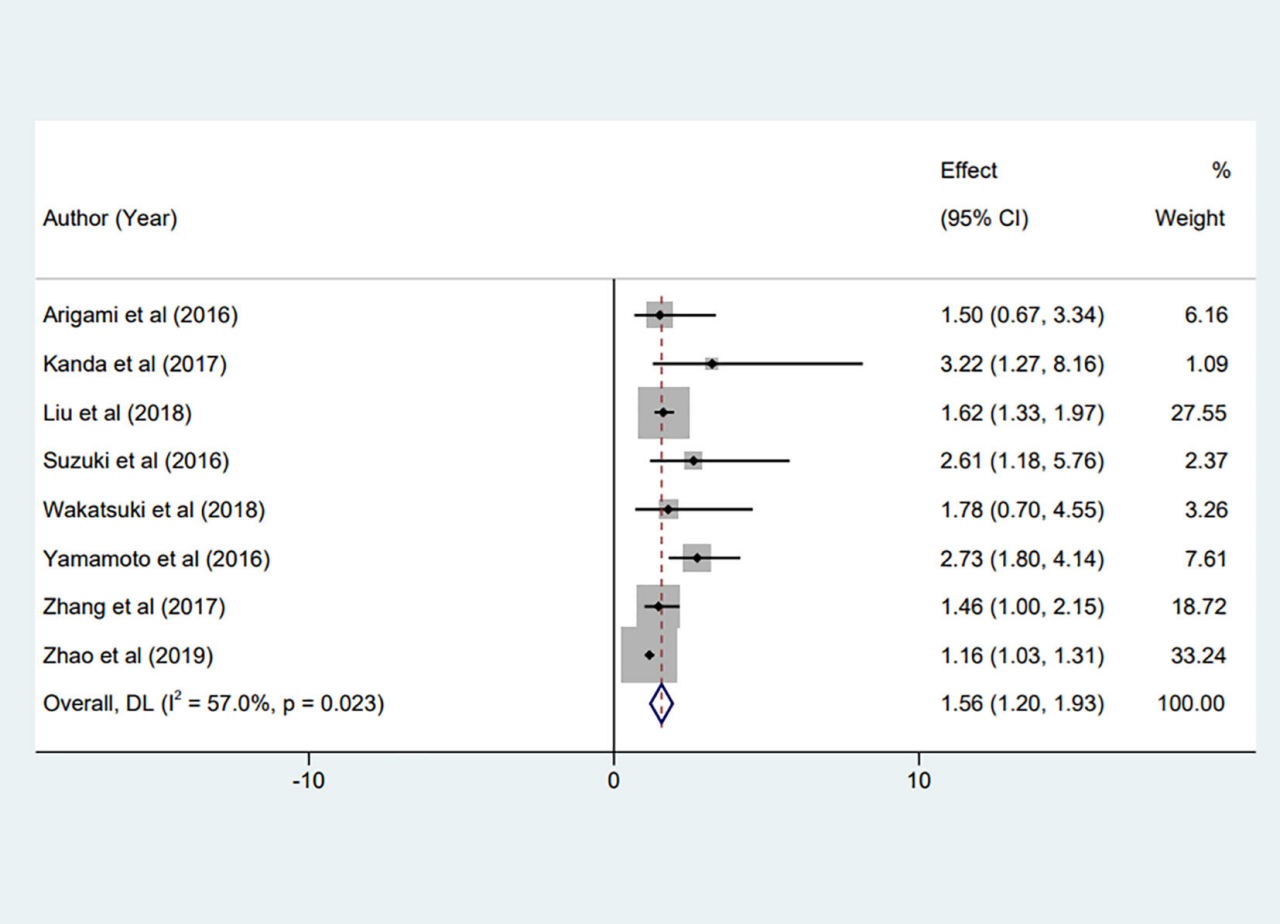
Forest plot of the meta-analysis on the relationship between preoperative Fib and patient OS.

Eight studies (17, 23–29), encompassing our meta-analysis, investigated the predictive value of preoperative fibrinogen levels for OS in patients with non-metastatic gastric cancer patients. The combined statistical analysis of HR derived from the multivariate analysis of OS revealed substantial heterogeneity across these studies (I² = 57.0%, P = 0.023). As a result, we employed a random-effects model for the analysis. Our findings indicated a significant correlation between elevated preoperative Fib levels and decreased OS in gastric cancer patients (HR = 1.56, 95% CI:1.20 to 1.93, P < 0.001), as illustrated in **Figure 2**.

### 3.5 Preoperative fibrinogen levels and RFS in gastric cancer

**Figure 3:**
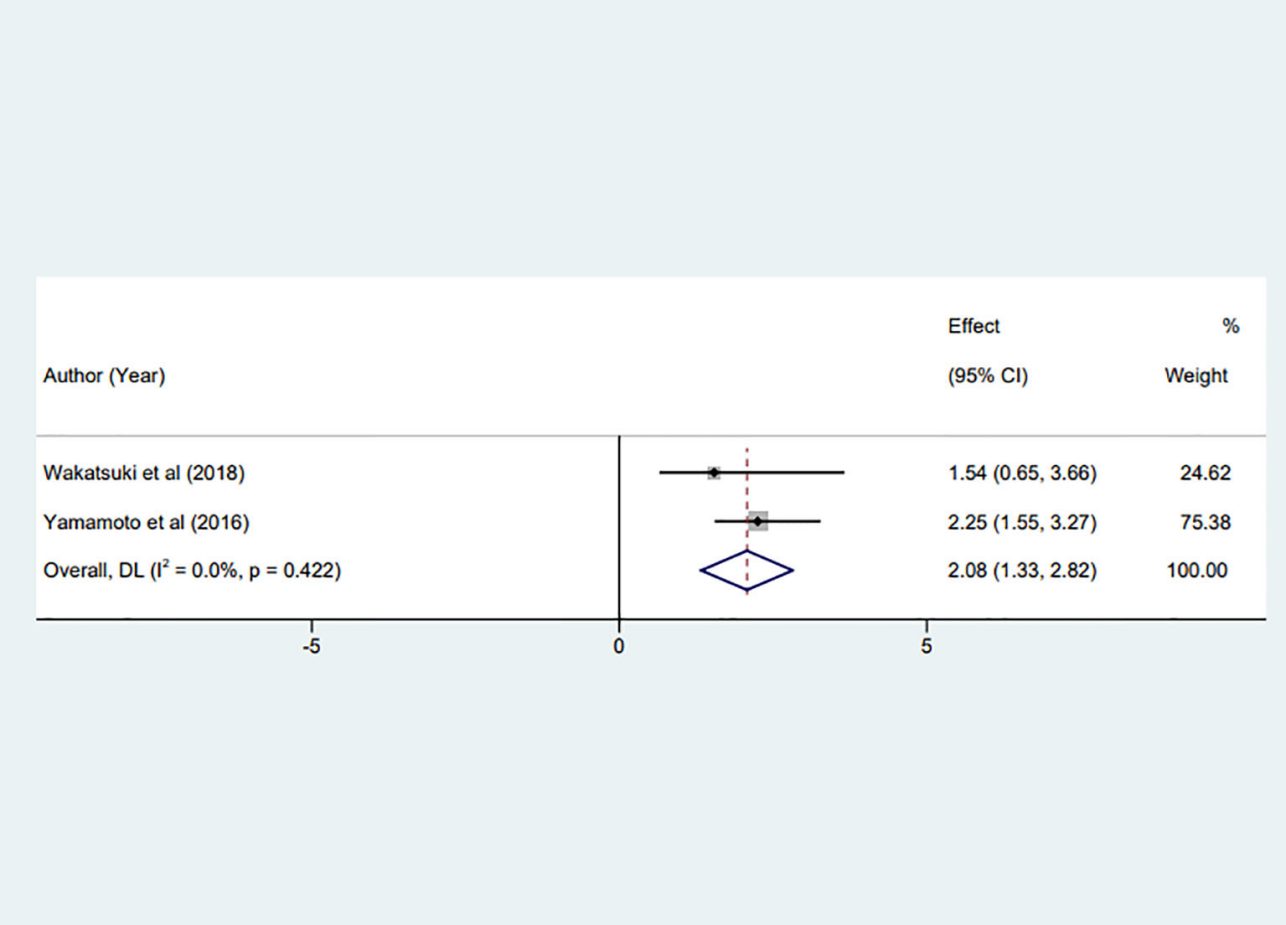
Forest plot of the meta-analysis evaluating the association between preoperative fibrinogen levels and recurrence-free survival (RFS) in gastric cancer, including two eligible studies.

Of the studies included in the meta-analysis, two discussed the prognostic value of preoperative fibrinogen levels for RFS in gastric cancer patients (17, 27). A combined statistical analysis of HR from the multivariate analysis of RFS demonstrated low heterogeneity between studies (I² = 0%, P = 0.422). Consequently, a fixed effects model was used. The analysis revealed a significant association between increased preoperative fibrinogen levels and diminished RFS in patients with gastric cancer (HR = 2.08, 95% CI:1.33 to 2.82, P < 0.001), as depicted in **Figure 3**.

### 3.6 Subgroup analysis

Subgroup analyses were performed to further explore the potential sources of heterogeneity and to validate the robustness of the association between preoperative Fib levels and OS. When stratified by country, elevated Fib was significantly associated with poorer OS in both Chinese (HR = 1.36, 95% CI: 1.15–1.80, P = 0.007) and Japanese populations (HR = 2.45, 95% CI: 1.85–3.30, P < 0.001). Analyses by sample size demonstrated consistent results, with significant associations observed in studies including fewer than 500 patients (HR = 1.77, 95% CI: 1.35–2.30, P < 0.001) and those with ≥500 patients (HR = 1.63, 95% CI: 1.16–2.40, P = 0.012). Regarding Fib cut-off values, patients with either lower cut-offs (<3.5 g/L, HR = 1.18, 95% CI: 1.09–1.32, P = 0.001) or higher cut-offs (≥3.5 g/L, HR = 2.20, 95% CI: 1.58–3.20, P < 0.001) consistently exhibited worse OS. Further subgroup analyses stratified by age, sex distribution, and tumor stage also supported the stability of the findings. Elevated Fib levels predicted inferior OS in both patients younger than 60 years (HR = 1.42, 95% CI: 1.12–1.85, P = 0.006) and those aged 60 years or older (HR = 1.61, 95% CI: 1.25–2.10, P < 0.001). Similarly, studies with male proportions <70% (HR = 1.47, 95% CI: 1.18–1.90, P = 0.003) and ≥70% (HR = 1.59, 95% CI: 1.22–2.05, P < 0.001) yielded comparable results. Collectively, these subgroup analyses reaffirmed the robustness of the association between elevated preoperative Fib levels and adverse OS across diverse demographic and clinical contexts (**Table 3**).

**Table 3:** Subgroup analysis of plasma fibrinogen levels and overall survival.

Subgroup analysis	Number of studies	HR (95% CI)	P for HR	I² (%)	P for I²	Model
Country						
China	3	1.36 (1.15 to 1.80)	0.007	68	0.03	Random
Japan	5	2.45 (1.85 to 3.30)	<0.001	0	0.67	Fixed
Sample size						
<500	5	1.77 (1.35 to 2.30)	<0.001	0	0.48	Fixed
≥500	3	1.63 (1.16 to 2.40)	0.012	89	<0.001	Random
Fib cut-off						
<3.5g/L	4	1.18 (1.09 to 1.32)	0.001	0	0.54	Fixed
≥3.5g/L	4	2.20 (1.58 to 3.20)	<0.001	62	0.04	Random
Age (median/mean)						
<60 years	3	1.42 (1.12–1.85)	0.006	42	0.15	Fixed
≥60 years	5	1.61 (1.25–2.10)	<0.001	38	0.18	Fixed
Sex (male proportion)						
<70% male	3	1.47 (1.18–1.90)	0.003	44	0.13	Fixed
≥70% male	5	1.59 (1.22–2.05)	<0.001	40	0.17	Fixed

### 3.7 Sensitivity analysis

**Figure 4:**
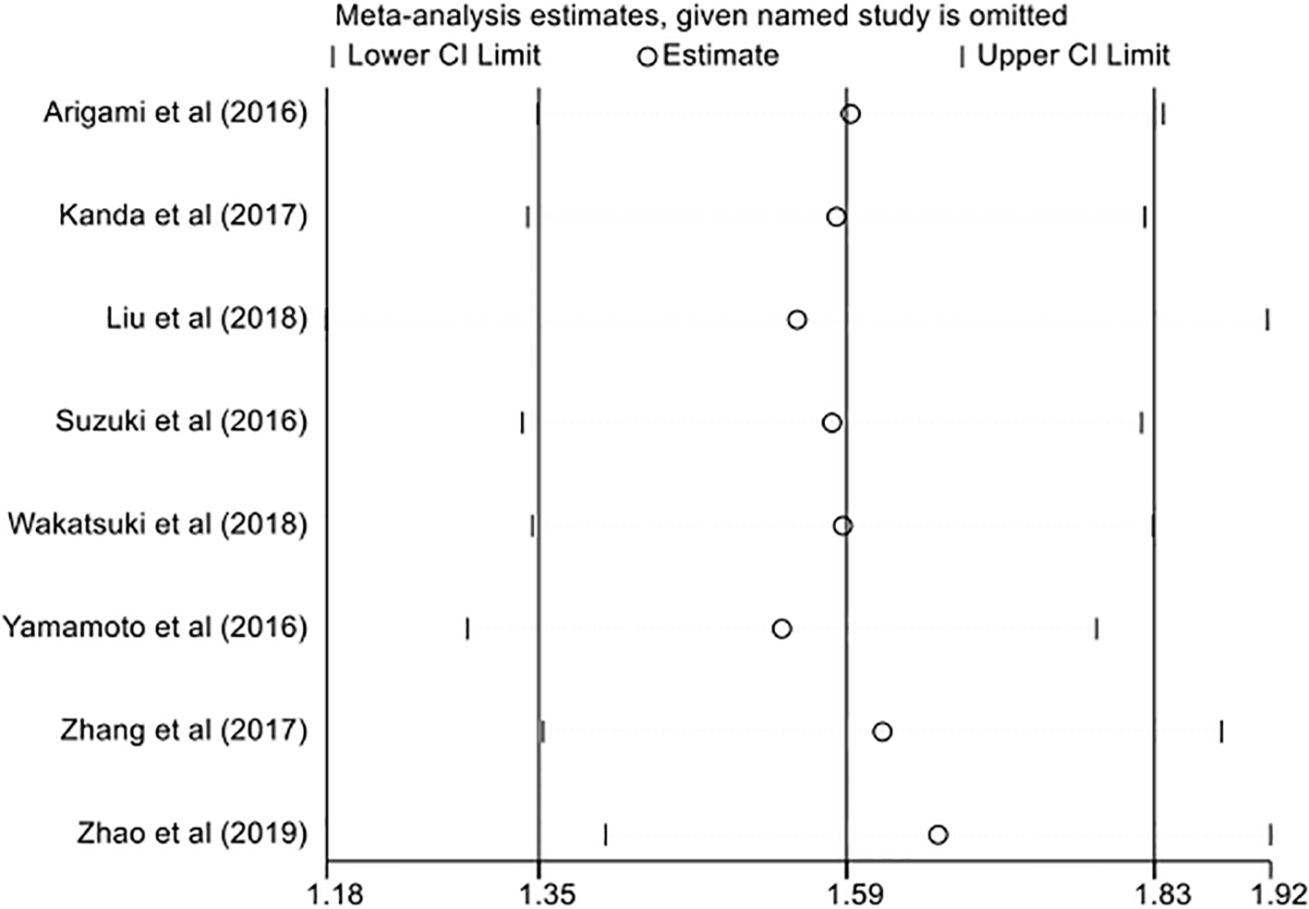
Sensitivity analysis of overall survival (OS) showing the effect of sequential omission of individual studies on the pooled hazard ratio estimates.

Given the significant heterogeneity among the included studies, we conducted leave-one-out sensitivity analyses to evaluate the stability and reliability of the pooled estimates. Each study was sequentially omitted, and the summary effect was recalculated using the remaining studies. The results were materially unchanged across iterations, indicating that no single study exerted undue influence on the overall findings. These consistent patterns bolster the credibility of our results and support the robustness of the primary outcomes (**Figure 4**).

### 3.8 Publication bias

**Figure 5:**
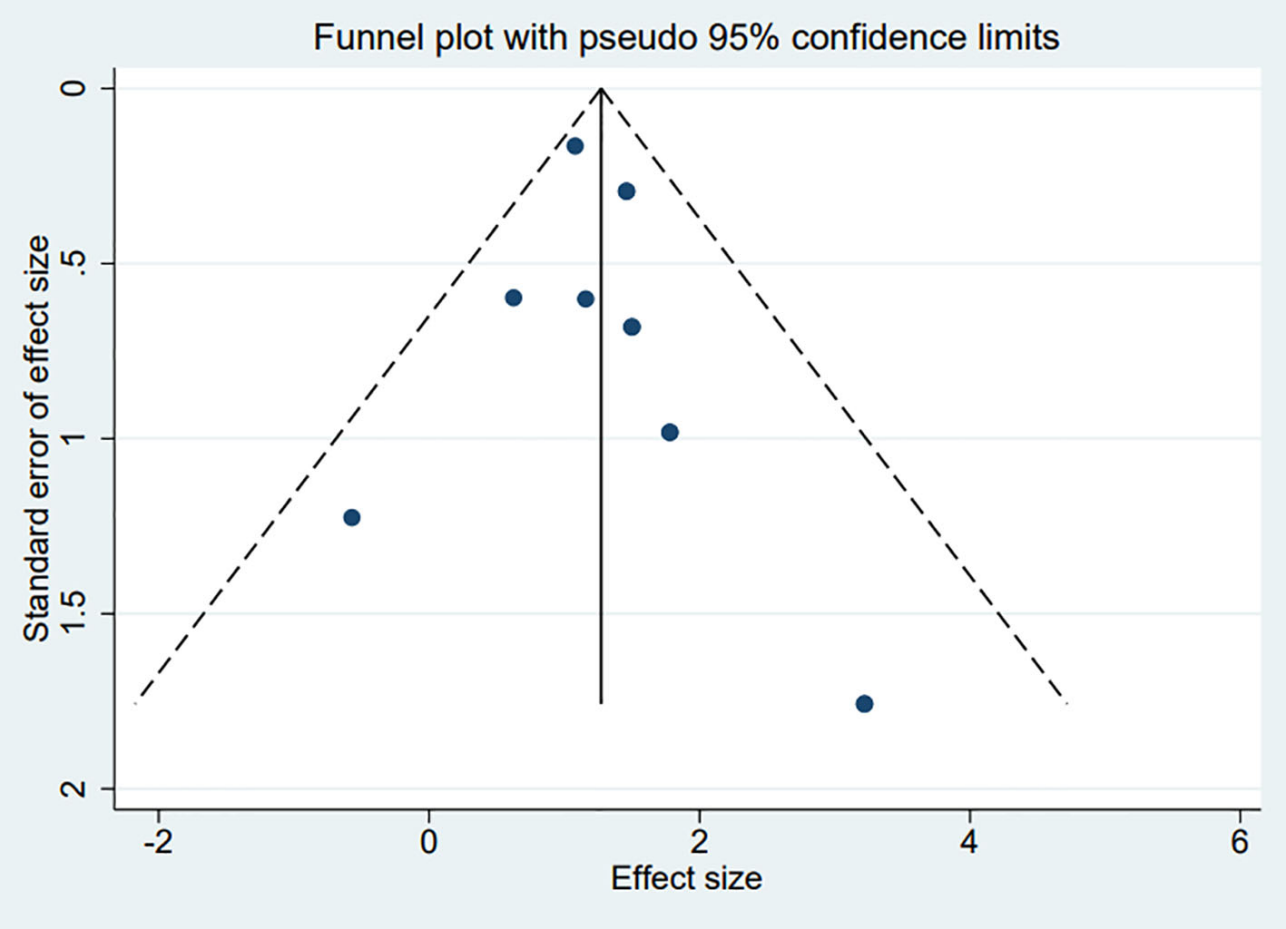
Funnel plot for publication bias in all included studies.

The funnel plots constructed in the observed study showed symmetry, and no significant publication bias was detected in the funnel plots (**Figure 5**). Egger’s linear regression test indicated that no significant publication bias was detected in the meta-analyses under different variables (P > 0.05), thus further confirming the robustness of the meta-analysis results.

## 4 Discussion

Fibrinogen is a hepatocyte-derived glycoprotein that participates in multiple physiological processes of hemostasis, including platelet aggregation, thrombin-mediated fibrin formation, and modulation of fibrinolysis. It also functions as a major acute-phase reactant, with circulating concentrations rising in response to systemic inflammation or tissue injury to help maintain homeostasis. Emerging evidence has increasingly linked elevated fibrinogen levels to adverse prognosis across several malignancies, including gastric cancer (30, 31). However, there remains a paucity of comprehensive meta-analyses specifically evaluating the association between preoperative plasma fibrinogen levels and outcomes in patients with non-metastatic gastric cancer.

A meta-analysis by Cheng et al. (32) provided preliminary insight into the prognostic role of fibrinogen in gastric cancer. However, their analysis included only four studies, some of which involved patients with metastatic disease, and the literature search was limited to February 2019, potentially omitting more recent evidence. To address this gap, the present study employed a comprehensive meta-analytic approach to objectively evaluate the prognostic significance of preoperative plasma fibrinogen levels in patients with non-metastatic gastric cancer. A total of eight retrospective studies comprising 3,999 patients were included. The pooled analysis demonstrated that elevated preoperative fibrinogen levels were significantly associated with poorer OS and RFS in patients with non-metastatic gastric cancer.

Nevertheless, the precise mechanisms underlying this association remain incompletely understood. Fib, a coagulation factor closely linked to inflammation, plays a pivotal role in the complex interplay between inflammatory responses and tumorigenesis (33). Previous studies have demonstrated that Fib can activate monocytes to secrete proinflammatory cytokines such as tumor necrosis factor-α (TNF-α) and IL-6, thereby promoting tumor cell proliferation and survival (34). In gastric cancer, patients often exhibit a hypercoagulable state, and tumor cells may stimulate Fib-mediated coagulation cascades, leading to thrombotic complications (35). Moreover, Fib contributes to tumor progression by sustaining angiogenic factors such as vascular endothelial growth factor and fibroblast growth factor, which play essential roles in cellular proliferation, invasion, and neovascularization (36). Zhang et al. (37) reported that elevated Fib levels may induce epithelial–mesenchymal transition (EMT) via activation of the p-AKT/p-mTOR signaling pathway, a key event in tumor invasion and metastasis. In addition, tumor cells frequently overexpress Fib receptors, and their interaction with Fib enhances tumor–host adhesion, facilitating tumor growth and dissemination (16). Concurrently, Fib promotes platelet aggregation by mediating β3-integrin activation in tumor cells, enabling immune evasion and increasing metastatic potential. Given that Fib measurement is simple, inexpensive, and widely accessible, combining Fib levels with conventional gastrointestinal tumor biomarkers could improve prognostic accuracy in gastric cancer patients (38). Such integrative approaches may also assist clinicians in designing more effective surveillance and follow-up strategies.

The moderate heterogeneity observed in the OS analysis (I² = 57%) likely reflects variability in fibrinogen cut-off values, sample sizes, and patient demographics or clinicopathological features across the included studies. To investigate potential sources of this heterogeneity, we performed extensive subgroup analyses stratified by country, sample size, cut-off threshold, age, sex distribution, and tumor stage. Notably, elevated preoperative fibrinogen levels remained significantly associated with poorer OS across all subgroups, indicating the robustness and consistency of this prognostic effect. Stronger effect estimates were observed in studies with higher cut-off values (≥3.5 g/L) and smaller sample sizes (<500), suggesting that methodological differences and population heterogeneity may contribute to between-study variance without altering the direction of association. Furthermore, the inclusion of mixed tumor stages (I–IV) in several studies may have introduced stratification bias, as stage is a major determinant of survival outcomes. Similar concerns were highlighted by Liu et al. (39), who demonstrated that heterogeneous stage composition could confound prognostic estimations in gastric cancer meta-analyses. From a biological perspective, higher fibrinogen cut-off thresholds may capture subpopulations with more pronounced systemic inflammation and hypercoagulability, reflecting intensified tumor–host interactions and pro-tumor immune modulation, which could explain their greater hazard ratios. This observation is also consistent with the oncologic inflammation paradigm, wherein elevated fibrinogen correlates with immunosuppressive microenvironmental remodeling and poorer survival. Supporting this view, Zhang et al. (40)reported that inflammation-associated biomarkers are closely linked to immune checkpoint dysregulation, further reinforcing fibrinogen’s role as an immune–coagulative mediator in cancer progression. Taken together, these findings suggest that the moderate heterogeneity is attributable to inherent clinical and methodological diversity, rather than inconsistency in prognostic impact. Thus, the association between elevated preoperative fibrinogen and adverse survival outcomes in non-metastatic gastric cancer appears robust across various analytic and biological contexts.

In clinical practice, the prognostic utility of fibrinogen could be enhanced through its integration with other systemic inflammatory biomarkers such as the neutrophil-to-lymphocyte ratio (NLR) and platelet-to-lymphocyte ratio (PLR) (38). Previous studies have shown that combined indices, such as the fibrinogen–NLR score (F-NLR), demonstrate superior predictive performance for survival outcomes in various malignancies compared to individual markers alone (41, 42). These composite scores capture both coagulation and immune-inflammatory dynamics, offering a more comprehensive reflection of tumor biology and host response. Thus, incorporating fibrinogen into multimodal prognostic models may improve risk stratification and guide individualized treatment decisions in gastric cancer patients. Emerging evidence has elucidated multiple biological mechanisms through which fibrinogen contributes to tumor progression. As an acute-phase reactant, fibrinogen is a key mediator linking systemic inflammation to oncogenesis. It promotes tumor cell proliferation and survival by interacting with IL-6 and other pro-inflammatory cytokines. Additionally, recent studies suggest that fibrinogen works synergistically with IL-8 in enhancing cytokine-driven immune modulation, further promoting cancer progression and immune escape (43). Furthermore, fibrinogen facilitates angiogenesis through the activation of vascular endothelial growth factor (VEGF), thereby enhancing nutrient supply to the tumor (44). Notably, fibrinogen can also promote EMT, a crucial process in tumor invasion and metastasis, by modulating extracellular matrix remodeling and interacting with integrins on tumor cells (45). In the context of hypercoagulability, fibrinogen’s role extends to metabolic pathways, including ferroptosis, a form of regulated cell death that influences tumor cell survival and immune modulation, as highlighted in recent work (43). These biological interactions underscore the multifaceted role of fibrinogen within the tumor microenvironment, supporting its prognostic significance in gastric cancer. Moreover, integrating fibrinogen with advanced imaging approaches, such as PET/CT radiomics, could offer multidimensional prognostic applications, providing a more holistic assessment of tumor dynamics and personalized treatment strategies (46).

Several limitations should be acknowledged. First, all included studies were conducted in China and Japan and primarily published in English, which may introduce language and regional selection biases, thereby limiting the generalizability of the findings. Second, the fibrinogen cut-off values varied across studies, potentially contributing to heterogeneity in the pooled estimates. Third, all studies were retrospective in design, which inherently raises concerns regarding selection bias and residual confounding, underscoring the need for prospective validation. Finally, the analysis of recurrence-free survival (RFS) was based on only two studies with relatively small sample sizes, limiting statistical power and precluding robust subgroup analyses. Although these studies showed consistent results with low heterogeneity, the findings regarding RFS should be interpreted cautiously. Future studies could consider integrating fibrinogen-based indices within systemic inflammatory scores and disulfidptosis molecular classifications to better capture the complex interplay between immune responses, oxidative stress, and tumor progression. Large-scale, multi-center prospective studies with standardized definitions of fibrinogen thresholds and comprehensive molecular profiling are warranted to confirm these associations and improve the clinical utility of fibrinogen as a prognostic biomarker in gastric cancer.

## 5 Conclusions

In conclusion, elevated preoperative fibrinogen levels are significantly associated with poorer OS and RFS in patients with non-metastatic gastric cancer, supporting its role as a potential prognostic biomarker. Given the limited number of studies reporting RFS outcomes, these findings should be interpreted with caution. Future large-scale, prospective investigations are needed to validate these associations and explore underlying biological mechanisms. Integration of fibrinogen into prognostic models may enhance risk stratification and inform individualized therapeutic strategies in gastric cancer management.

## Data Availability

The raw data supporting the conclusions of this article will be made available by the authors, without undue reservation.

## References

[1] Sung H Ferlay J Siegel RL Laversanne M Soerjomataram I Jemal A . Global cancer statistics 2020: GLOBOCAN estimates of incidence and mortality worldwide for 36 cancers in 185 countries. CA Cancer J Clin May. (2021) 71:209–49. doi: 10.3322/caac.21660, PMID: 33538338

[2] van den Berg YW Osanto S Reitsma PH Versteeg HH . The relationship between tissue factor and cancer progression: insights from bench and bedside. Blood. (2012) 119:924–32. doi: 10.1182/blood-2011-06-317685, PMID: 22065595

[3] Yu H Wang M Wang Y Yang J Deng L Bao W . The prognostic value of sarcopenia combined with preoperative fibrinogen-albumin ratio in patients with intrahepatic cholangiocarcinoma after surgery: A multicenter, prospective study. Cancer Med. (2021) 10:4768–80. doi: 10.1002/cam4.4035, PMID: 34105304 PMC8290250

[4] Ding P Wu J Wu H Ma W Li T Yang P . Preoperative liquid biopsy transcriptomic panel for risk assessment of lymph node metastasis in T1 gastric cancer. J Exp Clin Cancer Res. (2025) 44:43. doi: 10.1186/s13046-025-03305-x, PMID: 39915770 PMC11804050

[5] Janga LSN Sambe HG Yasir M Man RK Gogikar A Nanda A . Holistic understanding of the role of carbohydrate antigen 19–9 in pancreatic cancer screening, early diagnosis, and prognosis: A systematic review. Cureus. (2023) 15:e44382. doi: 10.7759/cureus.44382, PMID: 37671217 PMC10476147

[6] Passaro A Al Bakir M Hamilton EG Diehn M André F Roy-Chowdhuri S . Cancer biomarkers: Emerging trends and clinical implications for personalized treatment. Cell. (2024) 187:1617–35. doi: 10.1016/j.cell.2024.02.041, PMID: 38552610 PMC7616034

[7] Xie Y Liu F Wu Y Zhu Y Jiang Y Wu Q . Inflammation in cancer: therapeutic opportunities from new insights. Mol Cancer. (2025) 24:51. doi: 10.1186/s12943-025-02243-8, PMID: 39994787 PMC11849313

[8] Jing H Wu X Xiang M Wang C Novakovic VA Shi J . Microparticle phosphatidylserine mediates coagulation: involvement in tumor progression and metastasis. Cancers. (2023) 15:1957. doi: 10.3390/cancers15071957, PMID: 37046617 PMC10093313

[9] Nishida A Andoh A . The role of inflammation in cancer: mechanisms of tumor initiation, progression, and metastasis. Cells. (2025). doi: 10.3390/cells14070488, PMID: 40214442 PMC11987742

[10] Angelidakis E Chen S Zhang S Wan Z Kamm RD Shelton SE . Impact of fibrinogen, fibrin thrombi, and thrombin on cancer cell extravasation using *in vitro* microvascular networks. Adv Healthc Mater. (2023) 12:e2202984. doi: 10.1002/adhm.202202984, PMID: 37119127 PMC10524192

[11] Han L Yuan Y Feng Y Li X . Association of serum interleukin-6 and interleukin-8 levels with clinical benefit from immune checkpoint inhibitors in patients with advanced gastric cancer. Eurasian J Med Oncol. (2023) 7:227–31. doi: 10.14744/ejmo.2023.38141

[12] Ammendola M Vescio F Ammerata G Luposella M Patruno R Laface C . Ki-67 expression, mast cells positive to tryptase, and angiogenesis in gastric cancer patients undergoing radical surgery. Eurasian J Med Oncol. (2023) 7:62. doi: 10.14744/ejmo.2023.53961

[13] Wolberg AS . Fibrinogen and fibrin: synthesis, structure, and function in health and disease. J Thromb Haemost. Nov. (2023) 21:3005–15. doi: 10.1016/j.jtha.2023.08.014, PMID: 37625698 PMC10592048

[14] Song H Kuang G Zhang Z Ma B Jin J Zhang Q . The prognostic value of pretreatment plasma fibrinogen in urological cancers: A systematic review and meta-analysis. J Cancer. (2019) 10:479–87. doi: 10.7150/jca.26989, PMID: 30719143 PMC6360290

[15] Han L Yuan Y Feng Y Li X . Association of serum interleukin-6 and interleukin-8 levels with clinical benefit from immune checkpoint inhibitors in patients with advanced gastric cancer. EJMO. (2023) 7. doi: 10.14744/ejmo.2023.38141

[16] Yu X Hu F Yao Q Li C Zhang H Xue Y . Serum fibrinogen levels are positively correlated with advanced tumor stage and poor survival in patients with gastric cancer undergoing gastrectomy: a large cohort retrospective study. BMC Cancer. (2016) 16:480. doi: 10.1186/s12885-016-2510-z, PMID: 27418164 PMC4946212

[17] Wakatsuki K Matsumoto S Migita K Kunishige T Nakade H Miyao S . Prognostic value of the fibrinogen-to-platelet ratio as an inflammatory and coagulative index in patients with gastric cancer. Surg Today. (2019) 49:334–42. doi: 10.1007/s00595-018-1734-8, PMID: 30411155

[18] Page MJ McKenzie JE Bossuyt PM Boutron I Hoffmann TC Mulrow CD . The PRISMA 2020 statement: an updated guideline for reporting systematic reviews. Bmj. (2021) 372:n71. doi: 10.1136/bmj.n71, PMID: 33782057 PMC8005924

[19] Tierney JF Stewart LA Ghersi D Burdett S Sydes MR . Practical methods for incorporating summary time-to-event data into meta-analysis. Trials. (2007) 8:16. doi: 10.1186/1745-6215-8-16, PMID: 17555582 PMC1920534

[20] Parmar MK Torri V Stewart L . Extracting summary statistics to perform meta-analyses of the published literature for survival endpoints. Stat Med. (1998) 17:2815–34. doi: 10.1002/(sici)1097-0258(19981230)17:24<2815::aid-sim110>3.0.co;2-8, PMID: 9921604

[21] Zhou S Deng F Zhang J Chen G . Incidence and risk factors for postoperative delirium after liver transplantation: a systematic review and meta-analysis. Eur Rev Med Pharmacol Sci. (2021) 25:3246–53. doi: 10.26355/eurrev_202104_25733, PMID: 33928610

[22] Carra MC Romandini P Romandini M . Risk of Bias Evaluation of Cross-Sectional Studies: Adaptation of the Newcastle-Ottawa Scale. J Periodontal Res. (2025). doi: 10.1111/jre.13405, PMID: 40293188 PMC13665615

[23] Arigami T Uenosono Y Matsushita D Yanagita S Uchikado Y Kita Y . Combined fibrinogen concentration and neutrophil-lymphocyte ratio as a prognostic marker of gastric cancer. Oncol Lett. (2016) 11:1537–44. doi: 10.3892/ol.2015.4049, PMID: 26893776 PMC4734283

[24] Kanda M Tanaka C Kobayashi D Mizuno A Tanaka Y Takami H . Proposal of the coagulation score as a predictor for short-term and long-term outcomes of patients with resectab le gastric cancer. Ann Surg Oncol. (2017) 24:502–9. doi: 10.1245/s10434-016-5544-1, PMID: 27600621

[25] Liu X Liu Z Lin E Chen Y Sun X Zhou Z . A cumulative score based on preoperative fibrinogen and the neutrophil-lymphocyte ratio to predict outcomes in resectab le gastric cancer. Cancer Manag Res. (2018) 10:3007–14. doi: 10.2147/cmar.S174656, PMID: 30214295 PMC6118276

[26] Suzuki T Shimada H Nanami T Oshima Y Yajima S Ito M . Hyperfibrinogenemia is associated with inflammatory mediators and poor prognosis in patients with gastric cancer. Surg Today. (2016) 46:1394–401. doi: 10.1007/s00595-016-1339-z, PMID: 27160890

[27] Yamamoto M Kurokawa Y Miyazaki Y Makino T Takahashi T Yamasaki M . Usefulness of preoperative plasma fibrinogen versus other prognostic markers for predicting gastric cancer recurrence. World J Surg. (2016) 40:1904–9. doi: 10.1007/s00268-016-3474-5, PMID: 26969673

[28] Zhang J Li SQ Liao ZH Jiang YH Chen QG Huang B . Prognostic value of a novel FPR biomarker in patients with surgical stage II and III gastric cancer. Oncotarget. (2017) 8:75195–205. doi: 10.18632/oncotarget.20661, PMID: 29088857 PMC5650412

[29] Zhao LY Zhao YL Wang JJ Zhao QD Yi WQ Yuan Q . Is preoperative fibrinogen associated with the survival prognosis of gastric cancer patients? A multi-centered, propensity score-matched retrospective study. World J Surg. (2020) 44:213–22. doi: 10.1007/s00268-019-05191-9, PMID: 31637507

[30] Vilar R Fish RJ Casini A Neerman-Arbez M . Fibrin(ogen) in human disease: both friend and foe. Haematologica. (2020) 105:284–96. doi: 10.3324/haematol.2019.236901, PMID: 31949010 PMC7012490

[31] El-Sayed HA Sakr DH Abdelhakiem M Ebrahim MA Othman M Azzam H . Coagulation markers as independent predictors of colorectal cancer aggressiveness. BMC Gastroenterol. (2025) 25:634. doi: 10.1186/s12876-025-04249-4, PMID: 40898085 PMC12403284

[32] Cheng F Zeng C Zeng L Chen Y . Clinicopathological and prognostic value of preoperative plasma fibrinogen in gastric cancer patients: A meta-analysis. Med (Baltimore). (2019) 98:e17310. doi: 10.1097/md.0000000000017310, PMID: 31577724 PMC6783169

[33] Rafaqat S Gluscevic S Patoulias D Sharif S Klisic A . The association between coagulation and atrial fibrillation. Biomedicines. (2024) 12. doi: 10.3390/biomedicines12020274, PMID: 38397876 PMC10887311

[34] Greten FR Grivennikov SI . Inflammation and cancer: triggers, mechanisms, and consequences. Immunity. (2019) 51:27–41. doi: 10.1016/j.immuni.2019.06.025, PMID: 31315034 PMC6831096

[35] Marimuthu MMC Balamurugan BS Sundaram VA Anbalagan S Chopra H . Cytokine-based immunotherapy for gastric cancer: targeting inflammation for tumor control. Explor Target Antitumor Ther. (2025) 6:1002312. doi: 10.37349/etat.2025.1002312, PMID: 40309351 PMC12040674

[36] Ugwu AO Nnakenyi ID Okwor CJ . Investigating serum levels of IL-6 and TNF alpha, and the risk of thrombosis in newly diagnosed chemotherapy naïve obese cancer patients. Niger Med J. (2025) 66:761–9. doi: 10.71480/nmj.v66i2.845, PMID: 40703878 PMC12280287

[37] Zhang F Wang Y Sun P Wang ZQ Wang DS Zhang DS . Fibrinogen promotes Malignant biological tumor behavior involving epithelial-mesenchymal transition via the p-AKT/p-mTOR pathway in esophageal squamous cell carcinoma. J Cancer Res Clin Oncol. (2017) 143:2413–24. doi: 10.1007/s00432-017-2493-4, PMID: 28801734 PMC11819069

[38] Xue J Deng J Qin H Yan S Zhao Z Qin L . The interaction of platelet-related factors with tumor cells promotes tumor metastasis. J Transl Med. (2024) 22:371. doi: 10.1186/s12967-024-05126-6, PMID: 38637802 PMC11025228

[39] Liu Z Sun L Zhu W Zhu J Wu C Peng X . Disulfidptosis signature predicts immune microenvironment and prognosis of gastric cancer. Biol Direct. (2024) 19:65. doi: 10.1186/s13062-024-00518-6, PMID: 39148138 PMC11325698

[40] Zhang H Jiang Y Luo J Tan Q Xu M He B . Predictive and prognostic value of combined detection of sTim-3, PG and PD-L1 in immune checkpoint inhibitor therapy for advanced gastric cancer. Am J Transl Res. (2024) 16:6955–63. doi: 10.62347/mjoa5699, PMID: 39678619 PMC11645554

[41] Liu R Dai T Zheng S Deng M Lin G Bao Y . Prognostic value of combined pretreatment fibrinogen and neutrophil-lymphocyte ratio in digestive system cancers: a meta-analysis of 17 retrospective studies. Transl Cancer Res. (2021) 10:241–50. doi: 10.21037/tcr-20-2482, PMID: 35116256 PMC8797724

[42] Li X Zheng J Yan M Lu Y Pan X . The significance of fibrinogen in combination with the neutrophil to lymphocyte ratio in predicting the prognosis of patients with gastric cancer. Cancer Manag Res. (2022) 14:2313–21. doi: 10.2147/cmar.S374978, PMID: 35958950 PMC9359806

[43] Wu A Yang H Xiao T Gu W Li H Chen P . COPZ1 regulates ferroptosis through NCOA4-mediated ferritinophagy in lung adenocarcinoma. Biochim Biophys Acta (BBA) - Gen Subj. (2024) 1868:130706. doi: 10.1016/j.bbagen.2024.130706, PMID: 39181476

[44] Geindreau M Bruchard M Vegran F . Role of cytokines and chemokines in angiogenesis in a tumor context. Cancers (Basel). (2022) 14. doi: 10.3390/cancers14102446, PMID: 35626056 PMC9139472

[45] Prakash J Shaked Y . The interplay between extracellular matrix remodeling and cancer therapeutics. Cancer Discov. (2024) 14:1375–88. doi: 10.1158/2159-8290.Cd-24-0002, PMID: 39091205 PMC11294818

[46] Chen D Zhou R Li B . Preoperative prediction of her-2 and ki-67 status in gastric cancer using (18)F-FDG PET/CT radiomics features of visceral adipose tissue. Br J Hosp Med (Lond). (2024) 85:1–18. doi: 10.12968/hmed.2024.0350, PMID: 39347666

