# Supplementary material for: Relationship between preoperative plasma fibrinogen and prognosis in patients with non-metastatic gastric cancer: a systematic review and meta-analysis
Source: Front Oncol. 2025 Dec 4;15:1624602. doi: 10.3389/fonc.2025.1624602 (PMC12711540; doi:10.3389/fonc.2025.1624602)
Supplement: Supplementary file 1 [file Table1.docx]

**Supplementary Table S1:** Search strings utilised for the database search.

| Database | Search String |
| --- | --- |
| PubMed | (("Gastric Cancer"[Title/Abstract] OR "Stomach Cancer"[Title/Abstract] OR "Cardia Cancer"[Title/Abstract] OR "Gastric Neoplasm"[Title/Abstract] OR "Gastric Stomach Tumor"[Title/Abstract] OR "Tumor"[Title/Abstract] OR "Gastric Carcinoma"[Title/Abstract] OR "Stomach Carcinoma"[Title/Abstract] OR "Cardia Carcinoma"[Title/Abstract] OR "Cardia Tumor"[Title/Abstract]) AND ("fibrinogen"[Title/Abstract] OR "plasma fibrinogen"[Title/Abstract])) AND "prognosis"[Title/Abstract] |
| Embase | 'gastric cancer'/exp OR 'stomach cancer' OR 'cardia cancer' OR 'gastric neoplasm' OR 'gastric carcinoma' OR 'stomach carcinoma' OR 'cardia carcinoma' OR 'cardia tumor' AND ('fibrinogen'/exp OR 'plasma fibrinogen') AND 'prognosis'/exp |
| Web of Science | TS = (("Gastric Cancer" OR "Stomach Cancer" OR "Cardia Cancer" OR "Gastric Neoplasm" OR "Gastric Carcinoma" OR "Stomach Carcinoma" OR "Cardia Carcinoma" OR "Cardia Tumor") AND ("fibrinogen" OR "plasma fibrinogen") AND "prognosis") |
| Cochrane Library | ("Gastric Cancer" OR "Stomach Cancer" OR "Gastric Neoplasm" OR "Gastric Carcinoma") AND ("fibrinogen" OR "plasma fibrinogen") AND "prognosis" |
